# Effects of different starch structures on energy metabolism in pigs

**DOI:** 10.1186/s40104-023-00908-2

**Published:** 2023-08-09

**Authors:** Xiaoqian Gao, Bing Yu, Jie Yu, Xiangbing Mao, Zhiqing Huang, Yuheng Luo, Junqiu Luo, Ping Zheng, Hui Yan, Jun He, Daiwen Chen

**Affiliations:** Key Laboratory for Animal Disease-Resistance Nutrition, Ministry of Education, Institute of Animal Nutrition, Sichuan Agricultural University, Chengdu, 611130 People’s Republic of China

**Keywords:** Amylose, Amylopectin, Net energy, Pigs, Starch structure

## Abstract

**Background:**

Starch is a major component of carbohydrates and a major energy source for monogastric animals. Starch is composed of amylose and amylopectin and has different physiological functions due to its different structure. It has been shown that the energy supply efficiency of amylose is lower than that of amylopectin. However, there are few studies on the effect of starch structure on the available energy of pigs. The purpose of this study was to measure the effect of different structures of starch in the diet on the net energy (NE) of pigs using a comparative slaughter method and to establish a prediction equation to estimate the NE of starch with different structures. Fifty-six barrows (initial BW 10.18 ± 0.11 kg) were used, and they were housed and fed individually. Pigs were divided into 7 treatments, with 8 replicates for each treatment and 1 pig for each replicate. One of the treatments was randomly selected as the initial slaughter group (ISG). Pigs in the remaining treatments were assigned to 6 diets, fed with basic diet and semi-pure diets with amylose/amylopectin ratio (AR) of 3.09, 1.47, 0.25, 0.15 and 0.12, respectively. The experiment lasted for 28 d.

**Results:**

Results showed that compared with the high amylose (AM) groups (AR 3.09 and 1.47), the high amylopectin (AP) group (AR 0.15) significantly increased the final BW, average daily weight gain and average daily feed intake of pigs (*P* < 0.05), but the F:G of the AM group was lower (*P* < 0.01). In addition, AR 0.15 and 0.12 groups have higher (*P* < 0.01) nutrient digestibility of dry matter, crude protein, gross energy and crude ash. Meanwhile, compared with other groups, AR 0.15 group has a higher (*P* < 0.05) NE intake and energy retention (RE). The regressive equation for predicting with starch structures was established as RE = 1,235.243 − 48.298AM/AP (*R*^2^ = 0.657, *P* = 0.05).

**Conclusions:**

In conclusion, NE intake and RE of pigs augmented with the increase of dietary amylopectin content, indicating that diets high in amylopectin were more conducive to promoting the growth of pigs in the late conservation period.

## Background

Grain provides most of the energy in the pig diets, and starch, as the main component of grain feed, is the main energy source [1, 2]. Starch is mainly composed of α-glucans in the form of amylose and amylopectin. Amylose is considered to be a linear polysaccharide composed of α-*D*-glucose units linked by α-1,4-glycosidic bonds with less than 0.5% α-1,6-branching points [3]. Amylopectin is a larger, more branched molecule composed of α-*D*-glucose units linked by 95% α-1,4-glycosidic bonds and 5% α-1,6-glycosidic bonds [4]. Amylose/amylopectin ratio (AR) is the main factor affecting starch digestion [5]. Due to its molecular configuration and structure, amylose is not easy to digest in the small intestine, while amylopectin is easy to digest, which may lead to a rapid increase in postprandial blood glucose and insulin levels [6]. Undigested starch enters the large intestine with intestine peristalsis, where it is fermented by hindgut microorganisms to produce short-chain fatty acids (SCFA), which are then oxidized to provide 60%–70% of the energy for colonocytes [5]. Compared with glucose, SCFA is less efficient in converting into body energy. In the net energy (NE) system used to evaluate feed composition, the incremental efficiency of fermentable substrate converting to retained energy is generally assumed to be 70% of the enzymatically digestible starch [7, 8]. Therefore, we hypothesized that there might be differences in the energy efficiency of starch of different structure in the diet.

In some production systems, the cost of feeding can reach 80% of the total costs, considering the rise in the price of grains. Also, consider that within the fractions of the diet, energy is the proportion with the highest cost. However, compared with digestive energy (DE) and metabolic energy (ME) systems, NE system can more accurately reflect the actual available energy of pigs and maximize cost saving for pig producers [9]. There are two methods to determine NE: comparative slaughter method [10], indirect calorimetry [11]. In addition, the NE can also be calculated using the NE prediction equation based on chemical composition measurements of feeds and feedstuffs [12]. Because of the test equipment, the range of activity and food intake of experimental animals using indirect calorimetry were limited. For the chemical composition method, nutritionists do not have sufficient confidence in the coefficients in the equation, so this method has not been widely adopted. Therefore, the NE of the feed in the normal activity and diet of the animal cannot be presented. Thus, in large-scale pig production, the NE measured by slaughter method is more representative, which is known as the gold standard method for NE determination [12].

To the best of our knowledge, to date, it’s still relatively limited studies on the effects of diets with different AR on available energy use in pigs. Therefore, further research on this topic is needed. This experiment established the regression equation of NE by exploring the effect of different structural starch on pig's available energy. The regression equation can better predict the NE and reduce the feed cost. Furthermore, these results will provide theoretical data for future research on starch nutrition.

## Materials and methods

### Animal management and experimental design

Initially, fifty-six barrows weaned at 21 d were randomly divided into treatments according to average BW (10.18 ± 0.11 kg) and ADG after 2 weeks of pre-feeding. During pre-feeding, pigs were fed a corn-soybean meal based diet. Pigs were divided into 7 treatments, with 8 replicates per treatment and 1 pig per replicate. One group of pigs was randomly selected as the initial slaughter group (ISG), and all pigs in this group were slaughtered at the beginning of the experiment. The other 6 groups were assigned to 6 dietary treatments, and all pigs were slaughtered at the end of 28-d experimental period.

### Diets and feeding management

Following the NRC [13] recommendation for the nutrient requirements of 11–25 kg pigs, a corn-soybean meal based diet was formulated (Table 1). Starch diets were semi-pure diets made from corn starch with high amylose (76.79% amylose and 23.21% amylopectin) and high amylopectin (2.75% amylose and 97.25% amylopectin) in different proportions. The AR in the diets of each experimental group was 3.09, 1.47, 0.25, 0.15 and 0.12, respectively. The high amylose was purchased from Shanghai Quanwang Biotechnology Co., Ltd. (Shanghai, China) and the high amylopectin was purchased from Shandong Fuyang Biological Co., Ltd. (Dezhou, China). Diets were fed in mash form throughout the experiment. Chromium oxide was added to the diets (0.4%) from 24 d as an indigestible external marker for the determination of total tract nutrient retention by index method.

**Table 1:** Ingredient and chemical composition of experimental diets (as-fed basis)

Item	CON	AR				
		3.09	1.47	0.25	0.15	0.12
Ingredients, %						
Corn (7.8% crude protein)	27.00	-	-	-	-	-
Extruded corn	28.00	-	-	-	-	-
High amylose maize starch		53.00	44.00	17.00	10.00	-
High amylopectin maize starch			9.00	36.00	43.00	53.00
Soybean meal	12.57	13.11	13.11	13.11	13.11	13.11
Extruded soybean	6.70	6.50	6.50	6.50	6.50	6.50
Fish meal (62.5% crude protein)	4.50	6.00	6.00	6.00	6.00	6.00
Whey powder	6.00	5.00	5.00	5.00	5.00	5.00
Soy protein concentrate	6.40	11.00	11.00	11.00	11.00	11.00
Soybean oil	4.00	-	-	-	-	-
Sucrose	2.00	2.00	2.00	2.00	2.00	2.00
Cellulose	1.2	2.00	2.00	2.00	2.00	2.00
Limestone	0.60	0.44	0.44	0.44	0.44	0.44
Dicalcium phosphate	0.20	0.20	0.20	0.20	0.20	0.20
NaCl	0.20	0.20	0.20	0.20	0.20	0.20
*DL*-Lys·HCl (78%, crude protein)	0.10	-	-	-	-	-
*DL*-Met	0.03	0.05	0.05	0.05	0.05	0.05
Chloride choline	0.15	0.15	0.15	0.15	0.15	0.15
Vitamin and mineral premix^a^	0.35	0.35	0.35	0.35	0.35	0.35
Calculated content (as DM)^b^						
DE, Mcal/kg	3.60	3.70	3.70	3.70	3.70	3.70
Crude protein, %	19.76	19.62	19.62	19.62	19.62	19.62
Ca, %	0.70	0.70	0.70	0.70	0.70	0.70
Available P, %	0.33	0.33	0.33	0.33	0.33	0.33
*L*-Lysine, %	1.24	1.29	1.29	1.29	1.29	1.29
Methionine + cysteine, %	0.36	0.37	0.37	0.37	0.37	0.37
Threonine, %	0.80	0.62	0.62	0.62	0.62	0.62
Tryptophan, %	0.23	0.24	0.24	0.24	0.24	0.24
Total starch, % (measured values)	35.16	43.18	43.89	44.68	44.35	44.71
Amylose, % (measured values)	18.50	75.54	59.47	19.74	13.27	10.69
Amylopectin, % (measured values)	81.50	24.46	40.53	80.26	86.73	89.31

*CON* Control group, *AR* Amylose/amylopectin ratio

^a^Supplied (per kg diet): VA, 6,000 IU; VD_3_, 400 IU; VE, 10 IU; VK_3_, 2 mg; VB_1_, 0.8 mg; VB_2_, 6.4 mg; VB_6_, 2.4 mg; VB_12_, 12 µg; folic acid, 0.2 mg; nicotinic acid, 14 mg; *D*-pantothenic acid, 10 mg. Supplied (per kg diet): Fe as FeSO_4_·7H_2_O, 100 mg; Mn as MnSO_4_·7H_2_O, 4 mg; Zn as ZnSO_4_, 80 mg; Cu as CuSO_4_·5H_2_O, 6 mg; Se as Na_2_SeO_3_, 0.25 mg and I as KI, 0.3 mg

^b^Values are calculated composition except total starch, amylose and amylopectin contents

Each pig was individually housed in a metabolic cage (0.6 m × 1.7 m) with woven wire flooring in a temperature maintained at 27 ± 1 °C and the relative humidity was controlled at 65%–75% for the entire experiment. The pigs were fed 3 times every day (08:00, 14:00 and 20:00) during the whole experimental period. All animals were allowed ad libitum access to diets and water, and the leftovers were collected in time to avoided wasting.

### Collection of data and samples

The BW of pigs was recorded at the beginning of the experiment and at the end of each week. Feed intake was recorded daily to calculate ADG, ADFI and F:G at the end of the experiment.

From d 25 to 28, fresh fecal samples were collected and placed in separate plastic bags, and then 10 mL of 10% H_2_SO_4_ solution and a few drops of toluene were added per 100 g of fresh fecal samples to fix fecal nitrogen and prevent corrosion [14].

Comparative slaughter procedures were used to assess energy retention in pigs [15]. The body composition of 8 pigs slaughtered at the beginning of the experiment was measured and assumed to represent the initial body composition of all pigs in the experiment. Therefore, the initial body composition of each pig was calculated in the experiment and subtracted from the final body composition value to calculate the gain of protein, lipid and energy. Selected pigs were killed by intravenous administration of sodium pentobarbital (200 mL/kg, BW) [16]. Kil [17] described slaughter procedures and carcass measurement was used. Briefly, the carcass was split down the midline from the groin to the chest cavity and the visceral organs (stomach, intestine, liver, lungs, heart, kidneys and spleen) were removed and the carcass, viscera and blood were collected and weighed from each pig and processed separately. The carcass was divided into two equal parts and the right half was weighed and stored at −20 °C. The digestive tract was separated from other organs, emptied from digesta and weighed. The carcasses and viscera were ground to obtain representative subsamples and all subsamples of carcasses, viscera and blood were freeze-dried and finely ground prior to chemical analyses.

### Chemical analyses

Feces were oven dried at 65 °C for 72 h and finely ground to pass through 60-mesh screen before chemical analysis. The chromium contents was measured using an absorption spectrophotometer (HitachiZ-5000, Hitachi High-Technologies Corp., Tokyo, Japan) [18]. Determination of DM in diet and fecal samples by oven drying at 135 °C for 2 h [19]. DM of carcass, viscera and blood was calculated to constant weight by freeze-drying, and the value was used to calculate the energy, protein and fat concentration of the whole body. The GE of diets, feces, carcass, viscera and blood were measured using an adiabatic oxygen bomb calorimetry (Parr Instrument Co., Moline, IL, USA). Nitrogen in diets, fecal samples and in freeze-drying samples of body components was determined by a Kjeldahl procedure and protein was calculated as N × 6.25 (method 990.03, AOAC, 1995). Crude fat in diets, fecal samples and freeze-drying samples of body components was determined using the ether extraction method (method 945.16, AOAC, 1995). The ash in diet and fecal samples was determined (method 924.05, AOAC, 1995) [20].

Total starch and amylose were determined by assay kits (k-AMYL, Megazyme International Ireland Ltd., Wicklow, Ireland).

### Calculations

The apparent total tract digestibility (ATTD) of nutrients in the test diets were calculated using the index method [21]. The digestibility of chemical constituents was calculated using the following formula:

ATTD (%) = [1− (A1 × F2)/(A2 × F1)] × 100.

Where A1 = chromium content in diet, A2 = chromium content in feces, F1 = nutrient content in diet and F2 = nutrient content of feces [22]. The GE of the diet was multiplied by the ATTD of energy to calculate the DE of the diet at each diet. The total energy, protein and lipids per pig at slaughter were calculated from the sum of energy, protein and lipids in the blood, viscera and carcass. The energy, protein and lipids retained were calculated by subtracting the energy, protein and lipids in the pigs at the beginning of the experiment from the energy, protein and lipids in pigs at the conclusion of the experiment [23]. The initial body composition of the experimental pigs was determined by the body composition of the pigs in the ISG according to previously outlined procedures [23]. Energy retention was also calculated from the increase in protein and fat of 5.66 and 9.46 kcal/g, respectively [24]. The daily NE requirement for maintenance (NE_m_) for each pig was calculated by multiplying the mean metabolic BW (kg^0.6^) by 179 kcal [9]. The mean metabolic BW per pig was the average of the metabolic BW obtained each week during the 28-d experiment. The total NE for each diet was calculated by summing the NE_m_ and the RE in the body. The NE for starch diets were then calculated using the difference procedure by subtracting 47% of the NE in the CON from the NE calculated for starch diets [25].

### Statistical analysis

All data were analyzed by one-way analysis of variance (one-way ANOVA) in a completely randomized design using the General Linear Model (GLM) procedure of SPSS 22.0 (SPSS, Inc., Chicago, IL, USA). Duncan's multiple comparison test was used to distinguish statistical differences between treatments. Results were expressed as mean and SEM. *P* < 0.05 and *P* < 0.10 were considered statistically significant differences and a tendency to differ, respectively. Regression curve estimation was used to analyze the linear and quadratic relationships between ration starch structure and dependent variables.

## Results

### Growth performance and nutrient digestibility

The growth parameters are reported in Table 2. The initial BW did not differ among treatments; however, the final BW for pig fed the CON diet was lower (*P* < 0.05) than for pigs fed the semi-pure diets. For other 5 semi-pure diets, the pigs fed high amylose diets (AR 3.09 and 1.47) had lower final BW than those fed high amylopectin diets (AR 0.15 and 0.12). Similarly, pigs fed AR 0.15 diet had higher (*P* < 0.05) ADG, and ADFI than CON and high amylose groups. In addition, to our surprise, the AR 3.09 group had the lowest F:G (quadratic, *P* < 0.05).

**Table 2:** Effect of dietary starch structure on growth performance of pigs

Item	CON	AR					SEM	*P*-value		
		3.09	1.47	0.25	0.15	0.12		ANOVA	Linear	Quadratic
Initial BW, kg	10.07	10.10	10.14	10.13	10.24	10.28	0.12	0.996	0.586	0.860
Final BW, kg	25.48^b^	27.27^ab^	27.37^a^	27.81^a^	28.39^a^	27.63^a^	0.28	0.049	0.380	0.543
ADG, kg	0.55^b^	0.61^a^	0.62^a^	0.63^a^	0.65^a^	0.62^a^	0.01	0.015	0.448	0.522
ADFI, kg	0.92^b^	0.91^b^	0.96^ab^	1.00^ab^	1.05^a^	1.01^ab^	0.01	0.047	0.020	0.031
F:G, kg/kg	1.67^a^	1.49^c^	1.56^bc^	1.58^abc^	1.62^ab^	1.63^ab^	0.01	0.005	0.001	0.002

*AR* Amylose/amylopectin ratio, *CON* Control group, *ADFI* Average daily feed intake, *ADG* Average daily gain, *F:G* The ratio of average daily feed intake to average daily gain

^a–c^Means within a row with different superscripts significantly different (*P* < 0.05)

As shown in Table 3, increasing dietary amylopectin quadratically increased ATTD of DM, EE, GE, CP and ash (*P* < 0.01). Consistent with the growth performance, the nutrient digestibility of AR 0.15 group was the highest.

**Table 3:** Effect of dietary starch structure on carcass and body composition weight of pigs

Item	ISG	CON	AR					SEM	*P*-value
			3.09	1.47	0.25	0.15	0.12		
Live wt, kg	10.45	25.13	26.60	26.72	27.01	27.84	26.96	0.26	0.077
Hot carcass wt, kg	7.96	19.16^c^	19.55^bc^	19.94^abc^	20.81^ab^	21.47^a^	20.77^ab^	0.23	0.020
Dressing percentage, %	76.13	76.23^a^	73.43^b^	74.68^b^	77.02^a^	77.13^a^	77.01^a^	0.28	< 0.001
Chilled carcass wt, kg	7.86	18.97^c^	19.30^bc^	19.72^abc^	20.56^ab^	21.13^a^	20.51^ab^	0.22	0.025
Blood wt, kg	0.52	1.38	1.52	1.51	1.45	1.49	1.49	0.03	0.706
Full viscera wt, kg	1.03	2.69^b^	3.55^a^	3.27^a^	2.79^b^	2.78^b^	2.58^b^	0.07	< 0.001
Full viscera wt, % of live wt	9.82	10.73^b^	13.39^a^	12.23^a^	10.34^b^	10.01^b^	9.59^b^	0.24	< 0.001
Empty viscera wt, kg	0.82	2.03	2.18	2.05	2.02	1.89	1.96	0.04	0.455
Empty viscera wt, % of live wt	7.83	8.09^a^	8.20^a^	7.67^ab^	7.50^ab^	6.80^b^	7.27^ab^	0.15	0.009
Liver wt, kg	0.25	0.60^b^	0.65^ab^	0.65^ab^	0.65^ab^	0.70^a^	0.67^a^	0.01	0.033
Heart wt, kg	0.09	0.12	0.13	0.12	0.12	0.13	0.13	0.00	0.199
Kidney wt, kg	0.07	0.16	0.15	0.16	0.16	0.17	0.17	0.00	0.662
Lungs wt, kg	0.21	0.48	0.49	0.49	0.52	0.53	0.53	0.01	0.235
Spleen wt, kg	0.03	0.06	0.05	0.06	0.06	0.07	0.07	0.00	0.599
Total wt, kg	10.24	24.81	26.31	26.41	26.73	27.50	26.65	0.26	0.085

*AR* Amylose/amylopectin ratio, *CON* Control group, *ISG* Initial slaughter group, *wt* Weight, *Total wt* Sum of measured hot carcass weight, and the weight of blood, full viscera, liver, heart, kidney, lungs, and spleen

^a–c^ Means within a row with different superscripts significantly different (*P* < 0.05)

### Carcass composition and retention of energy, protein, and lipids

The body composition of initial and experimental pigs at slaughter are reported in Table 4. The high amylopectin groups tended to increase (*P* = 0.077) live weight and had higher (*P* < 0.05) carcass weights, dressing percentage and weight of all body components except the blood, full viscera weight and full viscera as a percentage of live weight.

**Table 4:** Effects of dietary starch structure on apparent total tract digestibility in pigs

Item	CON	AR					SEM	*P*-value		
		3.09	1.47	0.25	0.15	0.12		ANOVA	Linear	Quadratic
Dry matter	84.63^c^	82.46^d^	83.88^c^	88.82^b^	90.70^a^	89.59^ab^	0.49	< 0.001	< 0.001	< 0.001
Ether extract	78.51^a^	71.49^bc^	66.05^ cd^	60.20^d^	67.36^c^	76.44^ab^	1.24	< 0.001	0.221	< 0.001
Gross energy	85.42^c^	82.47^e^	83.86^d^	88.53^b^	90.80^a^	89.76^ab^	0.49	< 0.001	< 0.001	< 0.001
Crude protein	81.47^b^	70.35^c^	72.14^c^	83.34^ab^	85.26^a^	85.45^a^	0.97	< 0.001	< 0.001	< 0.001
Crude ash	47.92^ cd^	44.44^d^	45.16^d^	53.69^b^	61.31^a^	51.53^bc^	0.99	< 0.001	< 0.001	< 0.001

*AR* Amylose/amylopectin ratio, *CON* Control group

^a−e^Means within a row with different superscripts significantly different (*P* < 0.05)

The weight of the total digesta-free BW trended to increase (*P* = 0.063) with the increase of dietary amylopectin (Table 5). The total quantity of lipids, energy, the lipid gain:protein gain ratio, and measured energy retention increased linearly (*P* < 0.01) with increasing amylopectin, but protein gain was not affected by the content of dietary amylopectin. Lipid gain, lipid gain: protein gain ratio and calculated energy retention were greater (*P* < 0.05) for pigs fed AR 0.15 diet than for pigs fed the other 5 diets. In addition, there was no significant difference between the measured and calculated energy retention values.

**Table 5:** Effects of dietary starch structure on carcass composition, energy, protein and fat deposition in pigs

Item	ISG	CON	AR					SEM	*P*-value		
			3.09	1.47	0.25	0.15	0.12		ANOVA	Linear	Quadratic
Carcass composition											
DF BW, kg^1^	9.86	23.86	24.66	24.86	25.62	26.41	25.69	0.26	0.063	0.061	0.127
DF BW, kg DM	2.67	7.56	6.99	7.49	6.89	8.22	7.86	0.16	0.118	0.057	0.166
Total protein, kg	1.38	3.70	3.79	3.99	4.04	4.02	3.96	0.04	0.140	0.275	0.171
Total lipids, kg	0.70	2.74^ab^	2.17^c^	2.53^bc^	2.60^b^	3.10^a^	2.74^ab^	0.07	0.002	0.001	0.001
Total energy, Mcal	14.67	47.25^abc^	42.51^c^	46.34^bc^	46.96^abc^	51.92^a^	48.69^ab^	0.78	0.016	0.003	0.005
Retention											
Protein gain, g/d	-	82.81	86.15	93.24	95.17	94.46	92.12	1.60	0.137	0.272	0.168
Lipid gain, g/d	-	72.80^ab^	52.46^c^	65.37^bc^	67.97^b^	85.89^a^	72.81^ab^	2.44	0.002	0.001	0.001
Lipid:protein, g/g^2^		0.88^a^	0.61^c^	0.70^bc^	0.72^bc^	0.91^a^	0.79^ab^	0.02	< 0.001	0.001	0.001
MER, Mcal/d^3^	-	1.17^abc^	1.00^c^	1.13^bc^	1.15^abc^	1.33^a^	1.22^ab^	0.03	0.017	0.003	0.005
CER, Mcal/d^4^	-	1.16^abc^	0.98^c^	1.15^bc^	1.18^ab^	1.35^a^	1.21^ab^	0.03	0.009	0.003	0.002

*AR* Amylose/amylopectin ratio, *BW* Body weight, *CON* Control group, *ISG* Initial slaughter group

^1^DF BW = digesta-free BW, which is the sum of the weight of the chilled carcass, empty viscera, and blood

^2^Lipid:protein = the ratio of daily lipid gain to daily protein gain

^3^MER = measured energy retention

^4^CER = calculated energy retention (calculated from protein and lipid gain as 5.66 and 9.46 kcal/g for protein and lipid, respectively)

^a–c ^Means within a row with different superscripts significantly different (*P* < 0.05)

### NE of diets and supplemental starch

According to Table 6, the final quantity of energy, DE, energy retention and NE intake linearly increased (*P* < 0.05) as the content of dietary amylopectin increased. The final quantity of energy in the digesta-free body was greater (linear, *P* < 0.05) for pigs fed the AR 0.15 diet than for pigs fed the other 5 diets and compared to the other 5 groups, AR 0.15 group had the highest (linear, *P* < 0.05) energy retention and NE intake. However, there was no significant difference in NE of diets between different treatments (*P* > 0.05). The RE of the diet increased (*P* < 0.05) as dietary amylopectin increased and AR 0.15 group had the highest values. The RE of starch included at 43% amylopectin (1,399.60 kacl/kg) had the highest values (linear and quadratic, *P* < 0.01) than other 4 starch groups.

**Table 6:** Effect of dietary starch structure on net energy of pig diet

Item	CON	AR					SEM	*P*-value		
		3.09	1.47	0.25	0.15	0.12		ANOVA	Linear	Quadratic
Final body energy, Mcal	47.25^abc^	42.51^c^	46.34^bc^	46.96^abc^	51.92^a^	48.69^ab^	0.78	0.016	0.003	0.005
Energy retention, Mcal	32.58^abc^	27.84^c^	30.67^bc^	32.29^abc^	37.25^a^	34.12^ab^	0.78	0.016	0.003	0.005
Energy retention of starch, Mcal	-	23.64^c^	30.86^bc^	32.03^abc^	41.40^a^	35.29^ab^	1.68	0.010	0.003	0.005
DE, kcal/kg	3,519.12^a^	3,184.36^d^	3,219.62^d^	3,382.29^c^	3,447.86^b^	3,405.30^bc^	18.93	< 0.001	< 0.001	< 0.001
Total NE_m_, Mcal^1^	28.92	29.83	29.92	30.19	30.55	30.00	0.17	0.116	0.424	0.516
Total NE intake, Mcal^2^	61.50^ab^	57.66^b^	61.58^ab^	62.48^ab^	67.80^a^	64.02^ab^	0.92	0.040	0.008	0.012
Total feed intake, kg	25.68^b^	25.62^b^	26.98^ab^	27.93^ab^	29.36^a^	28.14^ab^	0.41	0.048	0.020	0.031
NE of diets, kcal/kg	2,399	2,260	2,291	2,247	2,313	2,274	22.95	0.465	0.772	0.949
NE of starch, kcal/kg^3^	-	2,138	2,195	2,112	2,236	2,162	43.98	0.923	0.775	0.950
RE of diets, kcal/kg	1,269^a^	1,082^b^	1,175^ab^	1,156^ab^	1,267^a^	1,206^ab^	19.01	0.029	0.013	0.024
RE of starch, kcal/kg	-	895.86^b^	1,134.65^ab^	1,136.23^ab^	1,399.60^a^	1,246.33^a^	46.91	0.008	0.002	0.004

*AR* amylose/amylopectin ratio, *CON* Control group, *DE* Digestible energy, *RE* Retained energy

^1^Total NE_m_ was calculated by multiplying the mean metabolic BW (kg^0.6^) of each pig by 179 kcal and the number of days the pigs were fed experimental diets

^2^Total NE intake = energy retention plus total NE_m_

^3^NE of starch = AR included at 53%; The NE of AR was calculated using the difference method by subtracting the NE contribution from the control diet from the NE of the diet containing AR

^a–c^ Means within a row with different superscripts significantly different (*P* < 0.05)

### Correlation between starch composition and RE of diets for pigs

According to Table 7, a high correlation coefficient was observed between AR and RE of diets (*r* = −0.810, *P* = 0.05). Amylopectin content tended to be positively correlated to RE of diets for pigs (*r* = 0.771, *P* = 0.073). In addition, there was a positive correlation between DE and RE of diets (*r* = 0.863, *P* = 0.027).

**Table 7:** The correlation coefficients between the chemical composition of the diet and the diets retained energy

Item	Correlation coefficient (*r*)	*P*-value
Dry matter	−0.359	0.484
Crude protein	−0.322	0.534
Ether extract	0.497	0.316
Ash	0.227	0.665
Gross energy	0.361	0.482
Digestive energy	0.863	0.027
Amylose	−0.771	0.073
Amylopectin	0.771	0.073
Amylose/Amylopectin	−0.810	0.050

### Prediction of RE of diets from the chemical composition of starch

According to Table 8, in order to obtain the best prediction with a simple linear regression equation, AM was used as the main parameter (Table 8, Eq. 1) and AR was used as the main parameter (Table 8, Eq. 2).

**Table 8:** Prediction of RE of diets (kcal/kg) from dietary chemical composition

No	Regressive equation	*R*^2^	RSD	*P-*value
1	RE = 1,235.243 – 48.298AM/AP	0.657	4.425	0.050
2	RE = 1,258.181 – 1.998AM	0.594	4.207	0.073

*RSD* Residual standard deviation, *RE* Retained energy, *AM* Amylose, *AP* Amylopectin, *TS* Total starch

## Discussion

With the development of the swine industry, it is inclined to use NE system to provide more accurate energy data to meet the needs of pig growth. Starch is the main source of energy in the swine diet, accounting for 55% of the diet, and its components (different AR) are closely related to the energy supply efficiency [26]. Although it has been shown that the energy supply value of amylose is lower than that of amylopectin [27], the NE value of different AR has not been determined. In this experiment, the NE value of different AR of dietary starch was determined to establish the NE prediction equation, reduce energy waste and save feed cost.

Our results showed that compared with the other five diets, the AR 0.15 diet increased the final BW, ADG and ADFI, and improve the F:G. These results are consistent with the finding of Fouhse et al. [28], which also indicated that pigs fed with the AR 0.15 diet grew faster and were more feed efficient than other 5 diets. However, our previous animal trials showed no effect on growth performance when piglets were fed diets with different AR [6]. This may be partly attributed to differences in pig age, with different stages having different nutritional needs and utilization. Due to the changes of environment, feed and immature development of digestive tract, weaned piglets cannot effectively utilize dietary nutrients, which will cause diarrhea and affect growth performance [29]. However, our preliminary study found that intestinal digestive function of piglets began to recover gradually at 2 weeks (d 35) after weaning (d 21), suggesting that piglets at the later stage of conservation were better able to utilize dietary nutrients than weaned piglets [30]. Among starch chemical characteristics, unlike highly branched amylopectin, amylose polymers have less surface area and more intra-molecular hydrogen bonds, resulting in less accessibility of the molecule to α-amylase and therefore, amylose polymers are digested at a lower rate and to lesser extent than amylopectin polymers [31]. Consistent with this, in vitro studies simulating the gastrointestinal environment of pigs have found that compared to high amylose, high amylopectin hydrolyzes faster and has a higher blood glycemic index [32]. To explore the effects of different AR of diets on nutrient utilization, ATTD was measured. Ingestion of AR 0.15 diet improved the digestibility of DM, CP, GE and Ash, which was consistent with the results of growth performance.

In this experiment, protein gain was not affected by the dietary AR. However, we were surprised to find that lipid gain and lipid:protein gain in the high amylopectin group was significantly higher than that in the high-amylose group. This result is also consistent with previous findings that feeding high amylose diets increases fat oxidation and reduces deposition [33, 34]. In addition, the high amylopectin group significantly increased the energy retention. Since the protein deposition rate was not affected, the difference in energy retention treatment was completely reflected in lipid retention, which was consistent with the results of rats [35] and pigs [27].

The DE of the high amylopectin diet (AR 0.15) was higher than that of high amylose diet (AR 3.09). A previous study also reported similar results in pigs [27] and ducks [36], which indicated that a diet with high amylose content may reduce DE and true metabolizable energy intake, respectively. In this study, we did not find that the starch structure of the diet had a significant effect on the NE of diets, but the high amylopectin diet had a numerical increase in the NE of diets compared with the high amylose diet. In this experiment, dietary treatments had no effect on NE_m_ of pigs, which may be part of the reason why dietary NE of diets did not differ significantly between treatments. Moreover, it is worth noting that the energy retention increases with the increase of amylopectin content. Previous experiments have also found that pigs fed a diet high in amylose produce less heat, making up for some of the energy loss [27, 37].

The correlation coefficients of DE, amylose (amylopectin), AR and RE of diets of feed samples in this experiment were 0.863, −0.711 (0.711) and −0.810, respectively. Correlation coefficients and prediction equation with chemical composition clearly show that amylose and AR composition mainly determine the energy value. This result is consistent with the research results of Zhou et al. [36], in which a prediction equation with *R*^2^ = 0.765 for true metabolizable energy was reported if the similar variables were used. Starch was an important predictor of dietary deposition energy, and similar results were obtained by Li et al. [38]. The reason may be that starch is an important component of the diet because of its high-energy value.

Overall, this experiment provides experimental data for the determination of NE in pigs on a large scale. High amylopectin can increase the RE and growth performance of pigs by increasing nutrient digestibility and fat deposition. Among them, the RE of AR 0.15 group was 185 kcal/kg higher than that of AR 3.09 group. In addition, further analysis of the energy value of starch with different structure found that fermented starch (AR 3.09) was 92%, 85% and 98% of DE, RE of diet and NE value of enzymatically degradable starch (AR 0.15), respectively.

## Conclusion

This study provides an in-depth understanding of the differences in energy supply of starch with different amylose/amylopectin ratios in diets, and shows that high amylopectin will increase the deposition energy in diets, especially fat deposition. It provides a theoretical basis for formulating accurate feed formula in the future and saves feed costs.

## Data Availability

The data analyzed during the current study are available from the corresponding author on reasonable request.

## Abbreviations

ADFI: Average daily feed intake
ADG: Average daily gain
AM: Amylose
AP: Amylopectin
AR: Amylose/amylopectin ratios
ATTD: Apparent total tract digestibility
BW: Body weight
DE: Digestive energy
F:G: Average daily feed intake:average daily gain
ISG: Initial slaughter group
ME: Metabolic energy
NE: Net energy
RE: Retention energy
SCFA: Short-chain fatty acids
